# State‐level estimates of excess hospitalizations and deaths associated with influenza

**DOI:** 10.1111/irv.12700

**Published:** 2019-11-07

**Authors:** Christopher A. Czaja, Lisa Miller, Kathryn Colborn, Myles G. Cockburn, Nisha Alden, Rachel K. Herlihy, Eric A. F. Simões

**Affiliations:** ^1^ Colorado Department of Public Health and Environment Denver CO USA; ^2^ Colorado School of Public Health Aurora CO USA; ^3^ University of Colorado School of Medicine Aurora CO USA

**Keywords:** death, hospitalization, human, influenza, public health

## Abstract

**Background:**

National estimates of influenza burden may not reflect state‐level influenza activity, and local surveillance may not capture the full burden of influenza.

**Methods:**

To provide state‐level information about influenza burden, we estimated excess pneumonia and influenza (P&I) and respiratory and circulatory (R&C) hospitalizations and deaths in Colorado from local hospital discharge records, death certificates, and influenza virus surveillance using negative binomial models.

**Results:**

From July 2007 to June 2016, influenza was associated with an excess of 17 911 P&I hospitalizations (95%CI: 15 227, 20 354), 30 811 R&C hospitalizations (95%CI: 24 344, 37 176), 1,064 P&I deaths (95%CI: 757, 1298), and 3828 R&C deaths (95%CI: 2060, 5433). There was a large burden of influenza A(H1N1) among persons aged 0‐64 years, with high median seasonal rates of excess hospitalization among persons aged 0‐4 years. Persons aged ≥65 years experienced the largest numbers and highest median seasonal rates of excess hospitalization and death associated with influenza A (H3N2). The burden of influenza B was generally lower, with elevated median seasonal rates of excess hospitalization among persons aged 0‐4 years and ≥65 years.

**Conclusions:**

These findings complement existing influenza surveillance. Periodic state‐level estimates of influenza disease burden may be useful for setting state public health priorities and planning prevention and control initiatives.

## 1 INTRODUCTION

Influenza has an enormous public health impact in the United States. The total economic burden of annual seasonal influenza epidemics has been estimated to be $87 billion.^1^ The Centers for Disease Control and Prevention (CDC) estimated there was an average of 24 000 influenza‐associated deaths per year and 64 influenza‐associated hospitalizations per 100 000 person‐years due to respiratory and circulatory causes in years prior to the 2009 influenza A(H1N1) pandemic.^2^, ^3^ The methods used to generate these burden estimates were developed to compensate for limitations in the frequency and accuracy of influenza diagnostic testing, factors that contribute to under‐detection of influenza‐associated outcomes by surveillance.^4^ CDC therefore estimates excess hospitalizations and deaths due to a range of diagnoses during periods of influenza circulation.^5^

However, national burden estimates may not specifically inform allocation of resources at the state or local public health level for prevention and response, and national rates may not reflect local populations or influenza circulation. The application of similar estimation methods to state‐level data has the potential to provide local burden estimates that complement existing influenza surveillance. We, therefore, adapted CDC’s methods and analogous methods from similar studies to estimate influenza excess hospitalizations and deaths in Colorado.^2^, ^3^, ^4^, ^5^, ^6^, ^7^, ^8^, ^9^, ^10^, ^11^, ^12^, ^13^, ^14^, ^15^, ^16^ We demonstrate that periodic disease burden estimates are feasible and provide additional information about serious influenza outcomes not routinely available in a state.

## 2 METHODS

### 2.1 Study design and study population

We estimated the burden of severe influenza in Colorado, a state with a population of 5.5 million people, from July 1, 2007, through June 30, 2016.^17^ Outcomes included influenza excess pneumonia and influenza (P&I) and respiratory and circulatory (R&C) hospitalizations and deaths. Estimates of these outcomes were obtained using regression models of weekly hospital discharges and deaths on circulating influenza over 469 weeks. The regression models identified numbers and rates of P&I and R&C hospitalizations and deaths during the influenza season that were in excess of a seasonal baseline. Population denominators were taken from the American Community Survey.^17^

### 2.2 Hospitalization and death data

To estimate influenza excess P&I and R&C hospitalizations and deaths, we first identified P&I and R&C hospitalizations and deaths recorded in statewide non‐federal acute care hospital discharge data and death certificate data provided by the Colorado Department of Public Health and Environment. P&I hospitalizations and deaths included records with a respective primary discharge diagnosis or underlying cause of death coded by ICD‐9 codes 480‐488 or ICD‐10 codes J09‐J18. R&C hospitalizations and deaths included records with a respective primary discharge diagnosis or underlying cause of death coded by ICD‐9 codes 390‐519 or ICD‐10 codes I00‐I99 or J00‐J99. These diagnoses groups were aggregated to weekly counts.

### 2.3 Viral surveillance data

The primary predictor of influenza excess P&I and R&C hospitalizations and deaths was the overall weekly percent of influenza tests in Colorado that were positive for influenza by type and subtype. These data were obtained from a representative sample of sentinel laboratories that provided influenza virus surveillance data to the Colorado Department of Public Health and Environment during the winter respiratory season from October through May of the next year. Tests were ordered at the discretion of the ordering physician and included a combination of molecular assays and antigen detection tests. State‐level data included the weekly number of tests performed and the number of tests positive for influenza A and B, without further subtyping of influenza A. Influenza A subtypes were assigned in proportion to the weekly ratio of H1N1 to H3N2 in CDC Region 8 (Colorado, Montana, North Dakota, South Dakota, Utah, and Wyoming).^3^, ^18^ We then calculated weekly percentages of influenza tests positive for A(H1N1), A(H3N2), and B.

We used single imputation to assign values for percent of influenza tests positive for weeks outside the period of seasonal surveillance. In general, this involved setting values for weeks in June through August to zero. However, the first wave of the influenza A (H1N1) pandemic occurred during the summer of 2009,^19^ during which time complete virus surveillance data were not available in Colorado. Therefore, for 15 weeks during the summer of 2009, values were imputed for influenza A (H1N1) using surveillance data from CDC Region 8. We created a simple linear regression model with Colorado values as the outcome and Region 8 values as the predictor during weeks for which we had a complete dataset, September 2009 – May 2010. We then stored the model coefficients and re‐ran the model to predict summer values in Colorado from available summer surveillance data from Region 8.

### 2.4 Time‐series regression models

**Figure 1:**
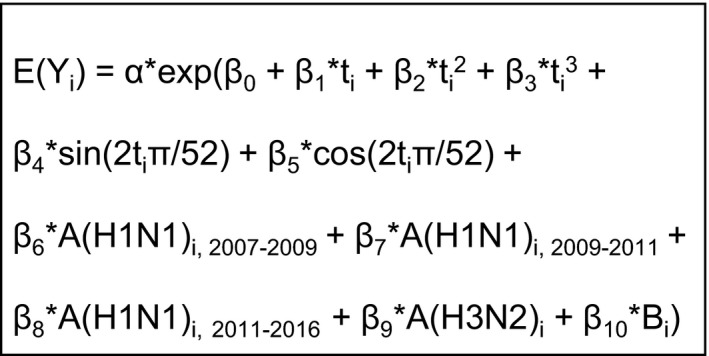
Negative binomial model used to estimate excess hospitalizations and deaths associated with influenza A(H1N1), A(H3N2), and B. E(Y_i_) is the expected number of hospitalizations or deaths at week i. α is the population offset taken from the American Community Survey 1‐year estimates assuming linear weekly growth between years.^17^ It is the number of the week in the time series. β_0_ is the intercept. β_1_ through β_3_ are coefficients associated with baseline linear, quadratic, and cubic time trends. β_4_ and β_5_ are coefficients associated with baseline seasonal changes. β_6_‐β_10_ are coefficients associated with percentages of specimens testing positive for each influenza virus type and subtype during a given week. Separate coefficients captured the effect of influenza A(H1N1) during pre‐pandemic, pandemic, and post‐pandemic periods. See the text for model fitting methods

Statistical analyses included a basic negative binomial model that was fit separately for P&I and R&C hospitalizations and deaths by age group (Figure 1). A negative binomial model was used to address over‐dispersion of data noted in Poisson models and confirmed by the LaGrange Multiplier test.^20^ In order to capture the changing association between circulating influenza A(H1N1) and hospitalization or death during pre‐pandemic, pandemic, and post‐pandemic time periods, the coefficients for influenza A(H1N1) were allowed to differ between July 1, 2007—June 30, 2009, July 1, 2009—June 30, 2011, and July 1, 2011—June 25, 2016 for most models. The pandemic period was extended to cover two influenza seasons for model stability and to capture immediate post‐pandemic activity separately from subsequent seasonal influenza. Exceptions included models of P&I and R&C hospitalizations for persons aged 0‐4 years which fit better with a single coefficient for influenza A (H1N1)_2007‐2016_. We constructed age‐stratified models to account for differences in the effect of circulating virus on hospitalizations or deaths by age group when possible; however, the ability to age‐stratify was limited by the number of events. Therefore, there were four models for each of P&I and R&C hospitalizations (0‐4 years old, 5‐49 years old, 50‐64 years old, and ≥65 years old), one model for P&I deaths (all ages combined), and two models for R&C deaths (<65 years old and ≥65 years old).

Model fitting incorporated the Akaike information criterion (AIC) and likelihood ratio tests.^20^ First, the basic model was constructed with lag periods of 0, 1, 2, or 3 weeks between virus surveillance findings and hospitalization or death, and AIC was used to select the best‐fitting lagged model (Table S1). The lag structure (0‐3 weeks) was held the same for each influenza type and subtype in an individual model. Next, the significance of β_1_‐β_3_ was evaluated using the combination of AIC and the likelihood ratio test, and corresponding variables were eliminated if they did not improve model fit.^20^ The remaining variables were forced into the model. Plots of observed versus predicted values demonstrated overlap to suggest model fit, and analyses of model residuals demonstrated no evidence of temporal autocorrelation, suggesting that the model adequately captured the seasonal baseline.

Excess P&I and R&C hospitalizations and deaths were defined as the difference in model‐predicted outcomes in the presence and absence of circulating influenza. These were obtained by taking the weekly predictions from the appropriate model and sequentially subtracting the weekly predictions from the same model applied to a dataset where circulation of the individual influenza type and subtype of interest was set to zero. The weekly differences between model predictions were summed over time. We estimated 95% confidence intervals (CI) from 1000 bootstrap samples of the data. Rate CIs were estimated using similar methods.^6^, ^17^ Count estimates and 95% CIs from age group‐specific models were summed to calculate total numbers and rates of influenza excess P&I and R&C hospitalizations and deaths.

The summary measures of primary interest included total counts and rates of influenza excess P&I and R&C hospitalizations and deaths and median seasonal rate estimates of the same. Medians were used rather than means because the data were positively skewed. Individual seasonal estimates were provided for context. We compared our estimates to the numbers of primary or underlying influenza‐specific diagnoses (ICD‐9 codes 487‐488 and ICD‐10 codes J09‐J11) captured from hospital discharge records and death certificates, respectively, and to the number of influenza hospitalizations captured by statewide laboratory‐based surveillance and those linked to death within 30 days. Analyses were conducted in SAS v. 9.4. The study was approved by the Colorado Multiple Institutional Review Board and the Institutional Review Board at the Colorado Department of Public Health and Environment.

## 3 RESULTS

### 3.1 Descriptive statistics for unmodelled hospitalizations, deaths, and virus surveillance

**Figure 2:**
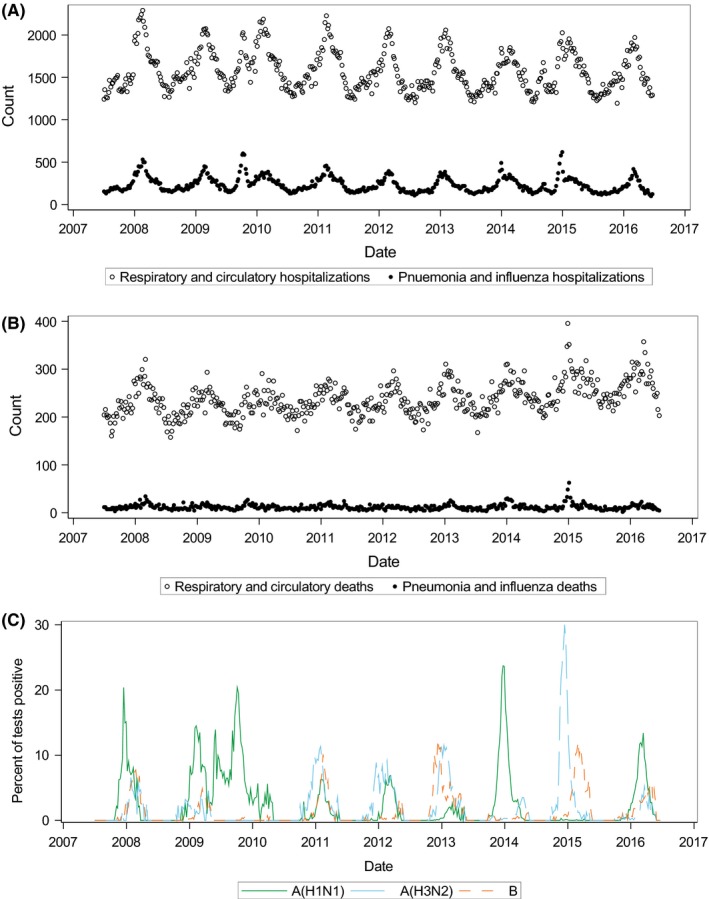
Time series of respiratory and circulatory (R&C) and pneumonia and influenza (P&I) hospitalizations from hospital discharge data (A), R&C and P&I deaths from death certificate data (B), and virus surveillance data from sentinel hospital laboratories (C), all ages—Colorado, July 1, 2007 through June 25, 2016

Between July 1, 2007, and June 25, 2016, there were 110 262 hospitalizations with a primary P&I discharge diagnosis and 738 307 hospitalizations with a primary R&C discharge diagnosis. There were 5582 deaths with a P&I diagnosis and 111 626 deaths with an R&C diagnosis as underlying cause of death. The age distribution of these recorded events is shown in Table 1. Time series of recorded hospitalizations and deaths demonstrated a seasonal pattern (Figure 2A‐B).

**Table 1:** Numbers of Recorded Diagnoses of Pneumonia and Influenza and Respiratory and Circulatory Hospitalizations and Deaths by Age—Colorado, 2007‐2016

Age	P&I^a^ Hospitalizations N = 110,262		R&C^b^ Hospitalizations N = 738,307		P&I^a^ Deaths N = 5,582		R&C^b^ Deaths N = 111.626	
	No.	%	No.	%	No.	%	No.	%
0‐4 y old	15 700	14.2	56 761	7.7	51	0.9	206	0.2
5‐49 y old	21 632	19.6	117 598	15.9	374	6.7	4585	4.1
50‐64 y old	19 533	17.7	172 478	23.4	709	12.7	15 732	14.1
≥65 y old	53 397	48.4	391 470	53.0	4,448	79.7	91 103	81.6

a P&I, pneumonia and influenza.

b R&C, respiratory and circulatory.

Time series of circulating influenza captured the 2009 pandemic and demonstrated the alternating predominance of influenza A(H1N1) and A(H3N2) (Figure 2C**)**. Influenza A(H1N1) was the predominant circulating virus in the 2007‐8 and 2008‐9 seasons. Pandemic A(H1N1) emerged during mid‐2009 and was predominant during the 2009‐10, 2013‐14, and 2015‐16 seasons. Influenza A(H3N2) circulated in other seasons and was predominant during the 2014‐15 season. Influenza B co‐circulated with influenza A viruses outside the 2009 pandemic. These sentinel laboratory surveillance data were used for all regression models.

### 3.2 Influenza excess P&I hospitalizations

There were an estimated 17,911 (95%CI: 15 227, 20 354) excess P&I hospitalizations during the 2007‐2008 through 2015‐2016 influenza seasons. The corresponding incidence rate was 38.72 per 100 000 person‐years (95%CI 32.92, 44.00). Over the study period, influenza A(H1N1) was associated with the largest number of excess P&I hospitalizations (9753; 95%CI: 9782, 10 623) (Table 2). By age group, influenza A(H1N1) was associated with the largest number of excess P&I hospitalizations among persons aged 0‐4 years, 5‐49 years, and 50‐64 years. Influenza A(H3N2) was associated with the majority of excess P&I hospitalizations among persons aged ≥65 years. Influenza B was associated with the second largest number of excess P&I hospitalizations among persons aged 0‐4 years.

**Table 2:** Estimated Total Numbers of Excess Hospitalizations and Deaths Associated with Influenza—Colorado, 2007‐2016

	A(H1N1)		A(H3N2)		B	
	No.	95% CI^a^	No.	95% CI^a^	No.	95% CI^a^
Hospitalizations						
Pneumonia & Influenza						
0‐4 y old	2153	(2941, 2401)	81	(−100, 261)	472	(294, 658)
5‐49 y old	3490	(3287, 3656)	573	(392, 737)	460	(246, 658)
50‐64 y old	2149	(1918, 2279)	981	(799, 1121)	613	(442, 790)
≥65 y old	1961	(1636, 2287)	4147	(3869, 4360)	831	(503, 1146)
All ages	9753	(9782, 10 623)	5782	(4960, 6479)	2376	(1485, 3252)
Respiratory & circulatory						
0‐4 y old	3043	(2626, 3461)	423	(77, 802)	1799	(1435, 2142)
5‐49 y old	4990	(4548, 5425)	101	(−309, 519)	1444	(1039, 1841)
50‐64 y old	3872	(3331, 4423)	818	(277, 1321)	1534	(999, 2046)
≥65 y old	2038	(1163, 2874)	7433	(6686, 8153)	3316	(2472, 4169)
All ages	13 943	(11 668, 16 183)	8775	(6731,10 795)	8093	(5945, 10 198)
Deaths						
Pneumonia & Influenza						
All Ages	457	(351, 534)	500	(394, 552)	107	(12, 212)
Respiratory & Circulatory						
0‐64 y old	633	(435, 792)	234	(69, 405)	137	(−56, 330)
≥65 y old	663	(249, 1028)	1514	(1104, 1817)	647	(259, 1061)
All ages	1296	(684, 1820)	1748	(1173, 2222)	784	(203,1391)

a 95% CI, 95% confidence interval.

Estimated median seasonal rates of excess P&I hospitalization demonstrated age group‐specific epidemiology by influenza type and subtype (Table 3). Median seasonal rates of influenza A(H1N1) excess P&I hospitalization were highest among persons aged 0‐4 years. Those for influenza A(H3N2) were highest among persons aged ≥65 years. Those for influenza B were relatively low with peaks among persons aged 0‐4 years and ≥65 years.

**Table 3:** Estimated Median Seasonal Rates of Hospitalization and Death Associated with Influenza—Colorado, 2007‐2016

	A(H1N1)		A(H3N2)		B	
	Rate^a^	95% CI^b^	Rate^a^	95% CI^b^	Rate^a^	95% CI^b^
Hospitalizations						
Pneumonia & Influenza						
0‐4 y old	75.20	(67.36, 84.24)	2.27	(−2.85, 7.29)	15.29	(9.70, 21.07)
5‐49 y old	7.49	(6.35, 9.07)	1.58	(1.08, 2.02)	1.38	(0.74, 1.96)
50‐64 y old	15.10	(13.73, 19.19)	9.11	(7.47, 10.36)	6.70	(4.80, 8.59)
≥65 y old	29.67	(24.61, 35.75)	67.03	(62.37, 70.26)	11.78	(7.19, 16.27)
Respiratory & Circulatory						
0‐4 y old	103.98	(88.79, 118.34)	9.97	(1.83, 18.76)	53.80	(43.18, 63.97)
5‐49 y old	9.86	(6.19, 12.89)	0.25	(−0.78, 1.29)	5.23	(3.77, 6.71)
50‐64 y old	35.68	(21.95, 39.87)	6.73	(2.26, 10.83)	18.36	(12.08, 24.50)
≥65 y old	18.75	(−0.10, 35.25)	112.89	(101.76, 123.92)	55.19	(41.11, 69.22)
Deaths						
Pneumonia & Influenza						
All Ages	0.78	(0.50, 1.12)	0.76	(0.59, 0.85)	0.23	(0.03, 0.46)
Respiratory & Circulatory						
0‐64 y old	1.24	(0.70, 1.89)	0.36	(0.11, 0.61)	0.40	(−0.16, 0.95)
≥65 y old	14.16	(2.72, 20.62)	20.52	(15.01, 24.80)	12.11	(4.85, 19.70)

a Rate per 100 000 persons.

b 95% CI, 95% confidence interval.

### 3.3 Influenza excess R&C hospitalizations

There were an estimated 30,811 (95%CI: 24 344, 37 176) excess R&C hospitalizations associated with influenza during the study period. The corresponding rate was 66.60 per 100 000 person‐years (95%CI 56.62, 80.36). Over the study period, influenza A(H1N1) was associated with the largest number of excess R&C hospitalizations (13 943; 95%CI: 11 668, 16 183) (Table 2). By age group, influenza A(H1N1) was associated with the largest number of excess R&C hospitalizations among persons aged 0‐4 years, 5‐49 years, and 50‐64 years. Influenza A(H3N2) was associated with the majority of excess R&C hospitalizations among persons aged ≥65 years. Influenza B was associated with the second largest number of excess R&C hospitalizations in all age groups.

Estimated median seasonal rates of excess R&C hospitalization demonstrated age group‐specific epidemiology by influenza type and subtype similar to that for P&I hospitalization but with a larger magnitude (Table 3). Median seasonal rates of influenza excess R&C hospitalization associated with A(H1N1) were highest among persons aged 0‐4 years. Those for influenza A(H3N2) were highest among persons aged ≥65 years. Those for influenza B were lower but peaked among persons aged 0‐4 years and ≥65 years.

### 3.4 Influenza excess P&I deaths

There were an estimated 1064 (95%CI: 757, 1298) excess P&I deaths associated with influenza during the study period. The corresponding rate was 2.30 per 100 000 person‐years (95%CI 1.64, 2.81). Influenza A(H1N1) and A(H3N2) were associated with the majority of excess P&I deaths with similar numbers by subtype (Table 2). Median seasonal rates for each were elevated relative to influenza B (Tables 3). Age group‐specific models of influenza excess P&I deaths could not be constructed due to the low number of events.

### 3.5 Influenza excess R&C deaths

There were an estimated 3828 (95%CI: 2060, 5433) excess R&C deaths associated with influenza during the study period. The corresponding rate was 8.27 per 100 000 person‐years (95%CI: 4.45, 11.74). Over the study period, influenza A(H3N2) was associated with the largest number of excess R&C deaths (1748, 95%CI: 1173, 2222) (Table 2). By age group, influenza A(H1N1) was associated with the largest number of excess R&C deaths among persons aged 0‐64 years, and influenza A(H3N2) was associated with the largest numbers of excess R&C deaths among persons aged ≥65 years. Influenza B was associated with a similar number of deaths among persons aged ≥65 years relative to influenza A(H1N1).

Estimated median seasonal rates of influenza excess R&C death were higher for persons aged ≥65 years compared to persons aged 0‐64 years for all influenza types and subtypes (Table 3). Among persons aged 0‐64 years, influenza A(H1N1) was associated with the highest median seasonal rate. Among persons aged ≥65 years, influenza A(H3N2) was associated with the highest median seasonal rate.

### 3.6 Rate estimates for individual seasons

**Figure 3:**
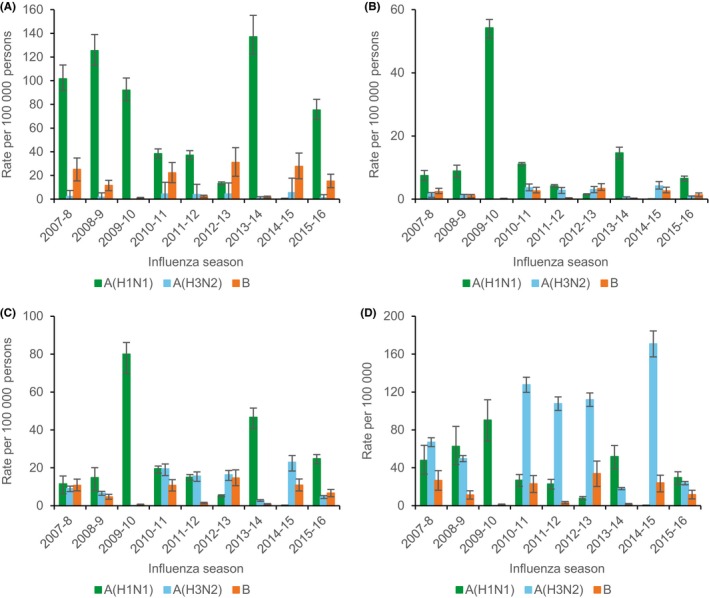
Estimated rates of excess pneumonia and influenza hospitalization associated with influenza for persons aged 0‐4 years (A), 5‐49 years (B), 50‐64 years (C), and ≥ 65 years (D) by influenza type and subtype and season—Colorado, 2007‐2009 through 2015‐2016 influenza seasons. Error bars indicate 95% confidence intervals. Note scale for y‐axis differs between panels

**Figure 4:**
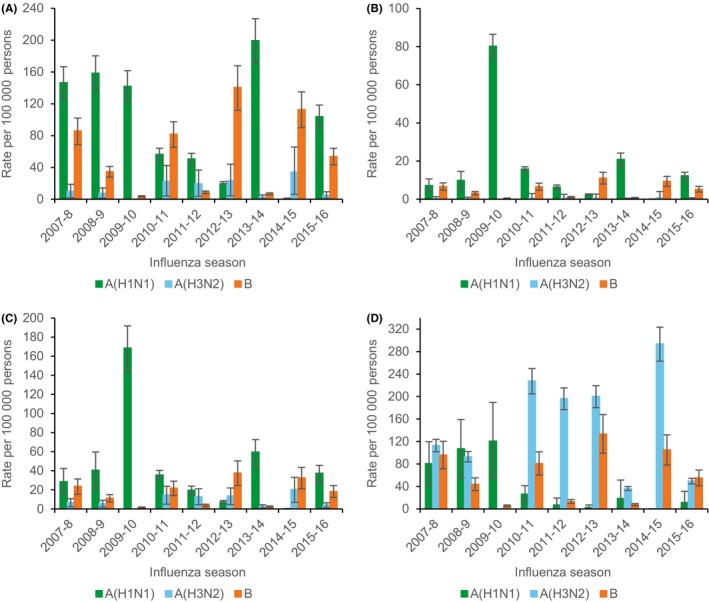
Estimated rates of excess respiratory and circulatory hospitalization associated with influenza for persons aged 0‐4 years (A), 5‐59 years (B), 50‐64 years (C), and ≥ 65 years (D) by influenza type and subtype and season—Colorado, 2007‐2009 through 2015‐2016 influenza seasons. Error bars indicate 95% confidence intervals. Note scale for y‐axis differs between panels

**Figure 5:**
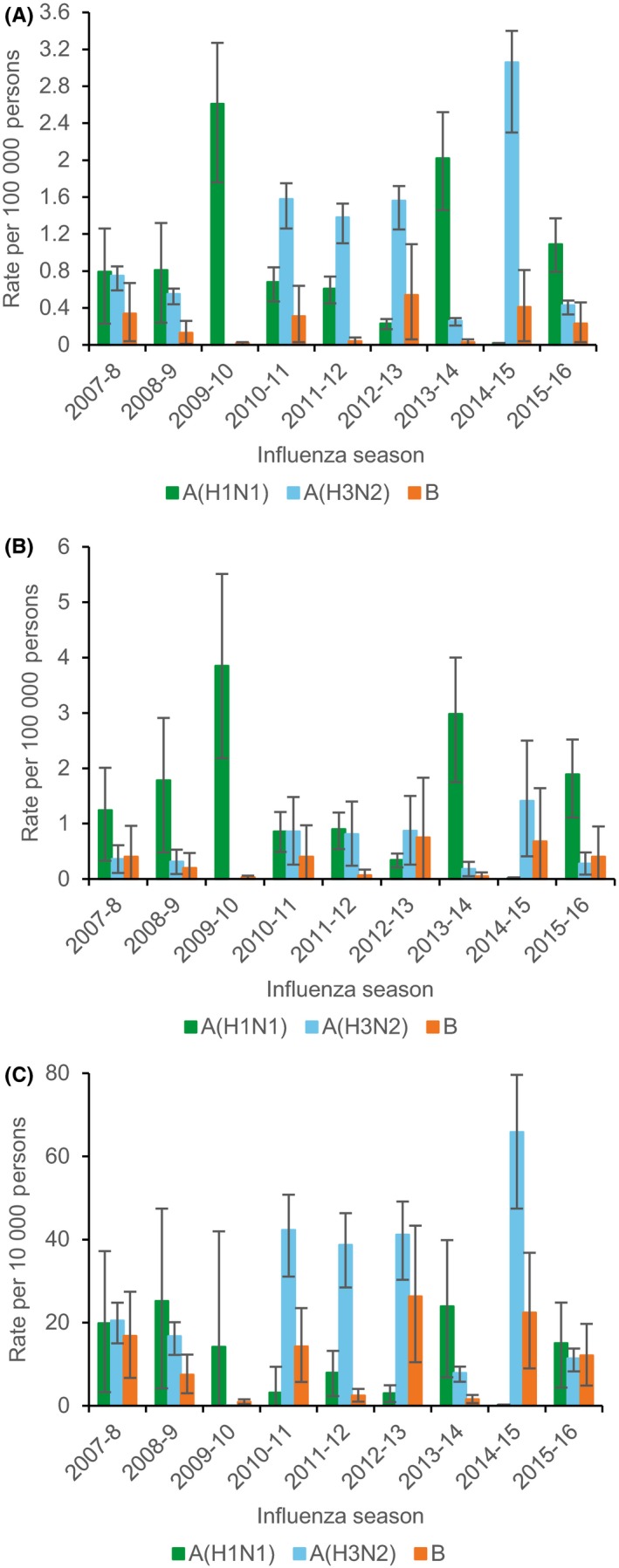
Estimated rates of excess pneumonia and influenza death, all ages (A), and respiratory and circulatory death for persons aged 0‐64 years (B) and ≥65 years (C) associated with influenza by influenza type and subtype and season—Colorado, 2007‐2009 through 2015‐2016 influenza seasons. Error bars indicate 95% confidence intervals. Note scale for y‐axis differs between panels

Estimates of rates of excess P&I and R&C hospitalization and death by season demonstrated year‐to‐year variability associated with predominant circulating influenza type and subtype and age, and the sporadic occurrence of unusually severe influenza seasons (Figures 3, 4, 5). In general, influenza A(H1N1) and A(H3N2) peaked in separate years. Rates of influenza A(H1N1) hospitalization and death dropped following the 2009‐2010 pandemic, peaked during the 2013‐2014 season, and were similar or greater than those for influenza A(H3N2) among children and adults aged <65 years during intervening years.

### 3.7 Evolution of influenza A(H1N1) over time

The model coefficients for percentages of specimens testing positive influenza A(H1N1) from surveillance differed during pre‐pandemic, pandemic, and post‐pandemic time periods, indicating that the association between virus circulation and influenza excess hospitalizations and deaths differed during these periods (Table S1). The greatest apparent differences occurred for excess P&I and R&C hospitalizations among persons 5‐49 years and 50‐64 years old. In these age groups, the coefficients were largest during the pandemic period, suggesting that pandemic influenza A(H1N1) was associated with higher rates of excess hospitalization and death than pre‐pandemic virus and that the rates of excess hospitalization and death associated with influenza A(H1N1) were lower in the post‐pandemic period. A similar pattern was not apparent in the models for excess P&I and R&C hospitalizations among persons ≥65 years old or in the models for P&I and R&C excess deaths, where the age groups were not as finely stratified.

### 3.8 Comparison to influenza‐specific diagnoses and laboratory‐confirmed influenza

Compared to hospitalizations and deaths with influenza recorded as the primary or underlying diagnosis, model estimates of total excess P&I hospitalizations, R&C hospitalizations, P&I deaths, and R&C deaths were 2.0, 3.4, 2.2, and 8.1 times higher, respectively (Table 4). There were similar discrepancies for each season.

**Table 4:** Comparison of influenza diagnoses recorded on hospital discharge records and death certificates, laboratory‐confirmed influenza hospitalizations and associated deaths within 30 d, and point estimates of influenza‐associated excess hospitalizations and deaths from regression models—Colorado, 2007‐2016

	Influenza diagnoses^a^	Model estimate, P&I^b^/influenza diagnoses^a^	Model estimate, R&C^c^/influenza diagnoses^a^		Laboratory‐confirmed influenza^d^		Model estimate, P&I^b^/laboratory‐confirmed influenza^d^		Model estimate, R&C^c^/laboratory‐confirmed influenza^d^	
	No.	Ratio	Ratio		No.		Ratio		Ratio	
Hospitalizations										
0‐4 y old	2016	1.3		2.6		2288		1.2		2.3	
5‐49 y old	2459	1.8		2.7		4320		1.0		1.5	
50‐64 y old	1506	2.5		4.1		2752		1.4		2.3	
≥65 y old	3194	2.2		4.0		5243		1.3		2.4	
Total	9175	2.0		3.4		14 603		1.2		2.1	
Deaths											
0‐64 y old	195	–e		5.1		205		–e		4.9	
≥65 y old	279	–e		10.1		388		–e		7.3	
Total	474	2.2		8.1		593		1.8		6.5	

a ICD‐9 codes 487‐488 and ICD‐10 codes J09‐J11 from hospital discharge records (hospitalizations) and death certificates (deaths).

b P&I, pneumonia and influenza.

c R&C, respiratory and circulatory

d Deaths within 30 d identified by linking laboratory‐confirmed hospitalizations to death certificates.

e –, no data.

Compared to laboratory‐confirmed influenza hospitalizations and linked deaths, model estimates of total excess P&I hospitalizations, R&C hospitalizations, P&I deaths, and R&C deaths were 1.2, 2.2, 1.8, and 6.5 times higher, respectively (Table 4). Individual seasonal estimates of excess P&I and R&C hospitalizations and deaths generally exceeded the numbers of laboratory‐confirmed influenza hospitalizations and linked deaths, except during the 2014‐2015 and 2015‐2016 seasons. During the latter seasons, the number of laboratory‐confirmed influenza hospitalizations was greater than the estimated number of excess P&I hospitalizations (2014‐15: 3541 vs 2056; 2015‐16: 1770 vs 1461).

## 4 DISCUSSION

National estimates of influenza burden do not provide state‐specific data for use by state and local public health agencies and may not reflect the underlying local population or circulation of influenza virus. We, therefore, used local and regional hospital discharge, death certificate, and influenza virus surveillance data to estimate numbers and rates of excess hospitalizations and deaths due to P&I and R&C causes associated with influenza over 9 influenza seasons in Colorado. The estimated numbers have the potential to inform allocation of public health resources. Notable findings during our study period included a predominant burden of influenza A(H1N1), especially among children and younger adults, a disproportionate burden of influenza A(H3N2) in older adults, and a substantial burden of influenza B. Estimated median seasonal rates depicted the local epidemiology of influenza hospitalization and death. We demonstrated median seasonal rates of excess hospitalization associated with influenza A(H1N1) among young children that were comparable to those associated with influenza A(H3N2) among older adults and moderately high median seasonal rates of excess hospitalization associated with influenza B at both extremes of age. Influenza B was associated with a median seasonal rate of excess R&C death among older adults comparable that for influenza A(H1N1) and lower than that for A(H3N2). These are sensitive estimates drawn from a wide range of diagnoses and many years of data. They complement ongoing influenza surveillance in Colorado, which captures influenza activity acutely through a variety of methods,^21^ and improve our understanding of an important but difficult‐to‐measure communicable disease.^4^, ^22^

We also captured the evolving epidemiology of influenza A(H1N1) over the years surrounding the 2009 pandemic. During 2009, the numbers and rates of excess hospitalizations and deaths attributed to influenza A(H1N1) were disproportionately high among persons aged <65 years, particularly among infants. The left shift in the age distribution of morbidity and mortality due to pandemic influenza A(H1N1) is thought to result from the presence of pre‐existing immunity among persons in older birth cohorts due to prior exposure to antigenically similar H1N1 viruses.^12^, ^15^, ^19^, ^23^, ^24^ In our study, influenza A(H1N1) continued to be associated with relatively high rates of excess hospitalization and death among the younger age groups when circulating during seasons following the pandemic. Time‐varying coefficients for influenza A(H1N1) in models for influenza excess P&I and R&C hospitalizations among persons 5‐49 years old and ≥65 years old increased in value during the pandemic period and decreased in value during the post‐pandemic period, suggesting a transient increase in virulence in these age groups consistent with prior pandemics.^25^

The above findings and our approach to estimating the state‐level burden of influenza apply to broader public health prevention and response efforts in several ways. First, we demonstrated that it is feasible to calculate state‐level estimates of influenza burden using methods established for national public health purposes.^2^, ^3^, ^4^, ^5^, ^6^, ^7^, ^8^, ^9^, ^10^, ^11^, ^12^, ^13^, ^14^, ^15^ Second, the approach accounted for the known under‐detection of influenza inherent to surveillance based on laboratory diagnosis or direct review of influenza diagnoses in administrative records.^4^ Our total count estimates exceeded the number of hospitalizations and deaths documented in hospital discharge and death certificate data by a factor of 2‐8, and the number of laboratory‐confirmed influenza hospitalizations and linked deaths by a factor of 1.2‐6.5, depending on outcome. Finally, the approach provided additional data not currently available from state‐level surveillance in Colorado. In particular, additional data included more detailed estimates of outcomes by influenza type and subtype, and more comprehensive information about influenza deaths. Such additional information provides a broader epidemiological context to existing surveillance findings.

While findings from this study reflect local influenza circulation and disease severity, consistencies with prior findings from studies using similar methods at the national level are reassuring. For example, in the United States from 1993 to 2008, the mean annual rate of influenza excess R&C hospitalization was 63.5 per 100 000 person‐years,^3^ whereas our rate for Colorado from 2007 to 2016 was 66.60 per 100 000 person‐years (95%CI 56.62, 80.36). In the United States from 1976 to 2007, the mean annual rate of influenza excess R&C death was 9.0 per 100 000,^2^ and ours was 8.27 (95%CI: 4.45, 11.74) per 100 000 person‐years for our study period. Our seasonal influenza type‐ and subtype‐specific rates are consistent with prior findings that influenza A(H1N1) causes more severe disease in younger age groups compared to influenza A(H3N2), which tends to exact a greater toll on persons aged ≥65 years.^2^, ^3^ Of note, the predominance of influenza A(H1N1) during the study period was reflected in higher estimates of influenza excess R&C hospitalization rates for A(H1N1) overall and by age group compared to national estimates from pre‐pandemic years.^3^ However, the comparability of our global state‐level estimates to prior national estimates attests to the validity of our results.

The best application of the regression modeling approach may be to supply periodic state‐level estimates of influenza burden in the form of total counts and median seasonal rates over several years. This is because the models generally require at least 5 years of data to be accurate.^5^ Furthermore, season‐specific estimates may be less accurate than overall estimates based on the full dataset, because regression models may not fully capture seasonal fluctuations in influenza virulence. This was evident in our study, where seasonal estimates of influenza excess P&I hospitalizations were lower than the number of laboratory‐confirmed influenza hospitalizations during the 2014‐2015 and 2015‐2016 seasons. Other investigators have addressed this issue by using separate model coefficients for influenza A for each season.^8^, ^11^, ^12^ Our attempts to do so were limited by power and the occurrence of negative estimates. While negative estimates are a known limitation to this approach, they must be interpreted with caution. For example, confidence limits that cross zero should be interpreted as an indicator of lack of statistical significance, rather than as a possible protective effect.^24^ To avoid misinterpretation, we allowed for the influenza A(H1N1) coefficient to change at biologically plausible time points coinciding with the emergence and evolution of the pandemic strain. Nevertheless, season‐specific influenza burden may be better estimated using other methods.^22^ It is also worthwhile noting that the interannual variability in influenza severity by season, type and subtype, and age will impact the overall summary measures.

A challenge to generating model estimates at the state‐level is to obtain accurate virus surveillance data. We did not have complete influenza A subtyping data from local surveillance, so we assigned influenza A subtypes based on regional proportions of H1N1 to H3N2. Others have applied regional virus surveillance data to state‐level outcomes.^13^ We imputed influenza A(H1N1) surveillance findings during the first wave of the 2009 pandemic, because year‐round surveillance data were not available. Other challenges may include lack of power to fit age‐stratified models for less frequent outcomes.

There are a few potential limitations to our study. One was the inability to account for respiratory syncytial virus (or other seasonal potential confounders) in our models, which may have led to overestimation in our results for influenza. However, other investigators have considered respiratory syncytial virus to be captured by the seasonal baseline.^5^, ^10^, ^15^ Another potential limitation was that influenza testing practices changed following the 2009 pandemic, which may have affected our virus surveillance data.^12^ Some authors have used additional indicators of influenza circulation, such as outpatient visits for influenza‐like illness, which we did not evaluate.^16^, ^26^ Finally, we did not compare our estimates to those extrapolated from surveillance for laboratory‐confirmed influenza hospitalization.^22^ Current United States methods of influenza disease burden estimation include surveillance for laboratory‐confirmed influenza hospitalization captured by the US Influenza Hospitalization Surveillance Network (FluSurv‐NET) and adjustment for under‐detection through the use of multipliers that account for testing practices and test characteristics. These methods were developed to create more timely national disease burden estimates that better capture season‐to‐season variation.^22^, ^27^ A potential future study would be to develop these newer methods for state‐level analyses and compare findings to state‐level estimates using the “Serfling‐type” models used in this study.^24^

In summary, there were nearly 18 000 excess hospitalizations and 1000 excess deaths due to P&I causes, and 31 000 excess hospitalizations and 4000 excess deaths due to R&C causes associated with influenza in Colorado over nine influenza seasons. These local estimates and corresponding rates provide additional information to state public health officials for setting priorities and planning interventions to prevent and control influenza. The methods are feasible and could be used to produce periodic reports of state‐level disease burden.
