# Supplementary material for: State‐level estimates of excess hospitalizations and deaths associated with influenza
Source: Influenza Other Respir Viruses. 2019 Nov 7;14(2):111–21. doi: 10.1111/irv.12700 (PMC7040963; doi:10.1111/irv.12700)
Supplement: Supplementary file 1 [file IRV-14-111-s001.docx]

| **Supplementary Table.** Lag structures and time-varying coefficients for viral surveillance from negative binomial model used to estimate excess hospitalizations and deaths associated with influenza A(H1N1). ^†^ | | | | | |
| --- | --- | --- | --- | --- | --- |
| Model | Lag Structure (Weeks) | β_2007-2009_ | β_2009-2011_ | β_2011-2016_ | p-value^ǂ^ |
| Hospitalizations |  |  |  |  |  |
| Pneumonia & Influenza |  |  |  |  |  |
| 0-4 Years Old | 2 | -^§^ | -^§^ | -^§^ | - |
| 5-49 Years Old | 1 | 0.0257 | 0.0957 | 0.0395 | <0.01 |
| 50-64 Years Old | 1 | 0.0134 | 0.0538 | 0.0464 | <0.01 |
| ≥65 Years Old | 1 | 0.0110 | 0.0155 | 0.0140 | <0.01 |
| Respiratory & Circulatory |  |  |  |  |  |
| 0-4 Years Old | 3 | -^¶^ | -^¶^ | -^¶^ | - |
| 5-49 Years Old | 1 | 0.0053 | 0.0301 | 0.0126 | <0.01 |
| 50-64 Years Old | 1 | 0.0044 | 0.0142 | 0.0078 | <0.01 |
| ≥65 Years Old | 2 | 0.0029 | 0.0026 | 0.0010 | 0.02 |
| Deaths |  |  |  |  |  |
| Pneumonia & Influenza |  |  |  |  |  |
| All Ages | 3 | 0.0164 | 0.0368 | 0.0347 | <0.01 |
| Respiratory & Circulatory |  |  |  |  |  |
| 0-64 Years Old | 1 | 0.0083 | 0.0138 | 0.0136 | <0.01 |
| ≥65 Years Old | 2 | 0.0032 | 0.0014 | 0.0035 | 0.03 |

^†^The same lag structure was used for influenza A(H3N2) and B within models.

^ǂ^Wald Χ^2^ test for change in the coefficient for influenza A(H1N1) surveillance over time (interaction term).

^§^β_2007-2016_ = 0.0386.

^¶^ β_2007-2016_ = 0.0158.
